# Decreased urinary excretion of norepinephrine and dopamine in autonomic synucleinopathies

**DOI:** 10.1007/s10286-024-01093-6

**Published:** 2024-12-10

**Authors:** David S. Goldstein, Patti Sullivan, Courtney Holmes

**Affiliations:** Autonomic Medicine Section (AMS), Clinical Neurosciences Program (CNP), Division of Intramural Research (DIR), National Institute of Neurological Disorders and Stroke (NINDS), National Institutes of Health (NIH), 10 Center Drive MSC-1620, Building 10 Room 8N260, Bethesda, MD 20892-1620 USA

**Keywords:** Dopamine, Norepinephrine, Kidney, Synuclein, Autonomic

## Abstract

**Background:**

Autonomic synucleinopathies feature autonomic failure and intracellular deposition of the protein alpha-synuclein. Three such conditions are the Lewy body diseases (LBDs) Parkinson’s disease (PD) and pure autonomic failure (PAF) and the non-LBD synucleinopathy multiple system atrophy (MSA). These diseases all entail catecholaminergic abnormalities in the brain, sympathetically innervated organs, or both; however, little is known about renal catecholaminergic functions in autonomic synucleinopathies. We measured urinary excretion rates of the sympathetic neurotransmitter norepinephrine, the hormone epinephrine, the autocrine-paracrine substance dopamine, the catecholamine precursor 3,4-dihydroxyphenylalanine (DOPA), 3,4-dihydroxyphenylglycol (DHPG, the main neuronal metabolite of norepinephrine), and 3,4-dihydroxyphenylacetic acid (DOPAC, a major dopamine metabolite), in PD, PAF, and MSA groups and controls.

**Methods:**

Data were reviewed from all research participants who had urine collections (usually 3.5 h) at the National Institutes of Health (NIH) Clinical Center from 1995 to 2024. The control cohort had neither autonomic failure nor a movement disorder.

**Results:**

Norepinephrine excretion rates were decreased compared with controls in PD (*p* = 0.0001), PAF (*p* < 0.0001), and MSA (*p* < 0.0001). Dopamine excretion was also decreased in the three groups (PD: *p* = 0.0136, PAF: *p* = 0.0027, MSA: *p* = 0.0344). DHPG excretion was decreased in PD (*p* = 0.0004) and PAF (*p* = 0.0004) but not in MSA. DOPA and epinephrine excretion did not differ among the study groups.

**Conclusions:**

Autonomic synucleinopathies involve decreased urinary excretion rates of norepinephrine and dopamine. Since virtually all of urinary dopamine in humans is derived from circulating DOPA, the low rates of urinary norepinephrine and dopamine excretion may reflect dysfunctions in the renal sympathetic noradrenergic system, the DOPA-dopamine autocrine-paracrine system, or both systems.

**Supplementary Information:**

The online version contains supplementary material available at 10.1007/s10286-024-01093-6.

## Introduction

The term, “autonomic synucleinopathies,” refers to a family of disorders characterized by intracytoplasmic deposition of the protein alpha-synuclein (α-syn) and symptoms or signs of autonomic failure. Examples of autonomic synucleinopathies are the Lewy body diseases (LBDs) Parkinson’s disease (PD) and the Lewy body form of pure autonomic failure (PAF), in which α-syn deposition is in LBs [36, 53]; and the non-LB synucleinopathy multiple system atrophy (MSA), in which α-syn deposition is in glial cytoplasmic inclusions [57]. PAF and PD can overlap neuropathologically [45], and clinically diagnosed PAF can evolve to PD or MSA [23, 37, 44].

Autonomic synucleinopathies entail abnormalities of central catecholaminergic systems. In addition to the well known striatal dopamine deficiency that characterizes PD [33] there are decreased tissue concentrations of norepinephrine throughout the brain in PD, including in the hypothalamus [12], putamen [27, 56], cerebellum [38], and cerebral cortex [52]. MSA entails prominent loss of catecholamine-synthesizing neurons in multiple brainstem regions [5–7, 11]. PAF has been reported to be associated with catecholamine deficiency in the brainstem but with sparing of striatal dopaminergic terminals [21].

Consistent with central catecholaminergic dysfunctions in autonomic synucleinopathies, cerebrospinal fluid (CSF) levels of norepinephrine and its metabolites 3,4-dihydroxyphenylglycol (DHPG) and 3-methoxy-4-hydroxyphenylglycol (MHPG) are decreased in PD, PAF, and MSA. Since levels of the dopamine metabolites 3,4-dihydroxyphenylacetic acid (DOPAC) and homovanillic acid are also decreased in PD and MSA but not in PAF [28], the three synucleinopathies seem to differ in the extents of central dopamine deficiency—prominent in PD and MSA, less apparent in PAF—but have in common central norepinephrine deficiency.

Autonomic synucleinopathies also feature catecholamine deficiencies outside the brain. This is especially apparent in the LB forms. An early finding was that in idiopathic orthostatic hypotension, renamed PAF [1], plasma levels of norepinephrine were low during supine rest, whereas they were normal in the Shy–Drager syndrome [60], renamed MSA [1, 2]. PD and PAF entail profoundly decreased tissue concentrations of both norepinephrine and dopamine in the left ventricular myocardium [25]. In these diseases peripheral noradrenergic deficiency is cardioselective [42, 55]. In contrast, MSA does not involve a post-ganglionic sympathetic lesion in most cases [19, 49], although there are exceptions [10, 47].

More than a half century ago, letters published in *Science* [4] and the *New England Journal of Medicine* [40] noted decreased urinary excretion of dopamine in PD. These and similar publications incited a line of research about whether the risk-free neurochemical approach of assaying catecholamines or their metabolites in urine might provide a means to identify central catecholamine deficiency in neuropsychiatric disorders. Research in neurology and psychiatry on plasma levels or urinary excretion of catecholamines and catecholamine metabolites continued for several years [14, 39, 43] before being largely abandoned [50]. At least one reason for the waning of interest was controversy evoked by reports about a “pink spot” in paper chromatographs from urine of patients with schizophrenia [3, 16] or PD [8]. The pink spot was proposed to represent a pathogenic metabolite of dopamine, 3,4-dimethoxyphenylethylamine; however, efforts to replicate the findings failed, and evidence accrued instead for excretion of tyramine [8, 32], which is not endogenous in humans.

Subsequently it was found that essentially all of urinary dopamine in humans is derived not from the brain or even from nerves but from renal uptake and decarboxylation of circulating DOPA [59]. Circulating DOPA in turn has multiple sources, including the diet [29, 58], sympathetic nerves [30], and parenchymal cells in splanchnic organs [13].

Recently there has been a revival of interest in the possibility of dysfunctional peripheral catecholaminergic systems in autonomic synucleinopathies because of evidence that the disease processes may begin outside the brain, with early involvement of the autonomic nervous system [22, 34]. These findings prompted us to revisit the topic of urinary excretion of norepinephrine, dopamine, their deaminated metabolites, and the catecholamine precursor 3,4-dihydroxyphenylalanine (DOPA) in PD, PAF, and MSA.

Whether urinary excretion rates of norepinephrine and its main neuronal metabolite, DHPG, are decreased in autonomic synucleinopathies has been unknown, despite reports indicating at least some degree of renal noradrenergic deficiency in PD and PAF [42, 55]. Whether excretion of norepinephrine, dopamine, and other 3,4-dihydroxy compounds (catechols) are correlated across patients has also been unknown.

This retrospective, observational study therefore examined urinary excretion rates of the catechols norepinephrine, dopamine, DHPG, DOPAC, epinephrine, and DOPA in groups of patients with autonomic synucleinopathies.

## Methods

Data and samples from the National Institute of Neurological Disorders and Stroke (NINDS) institutional review board (IRB)-approved protocols 94N0186, 03N0004, and 18N0140 were transferred to the secondary research protocol 000490, “Mechanisms of Autonomic and Catecholamine-Related Disorders,” and the study reported here was approved by the National Institutes of Health (NIH) IRB as an amendment to the 000490 protocol. All participants had been tested at the NIH Clinical Center after giving written informed consent. Follow-up testing was per the protocols of the original studies. For comparison purposes, data were also reviewed from patients referred for comprehensive autonomic function testing who did not have evidence for sympathetic neurocirculatory failure or a movement disorder.

Urine collections were obtained in conjunction with ^18^F-dopamine positron emission tomography (PET), beginning before injection of the tracer (usually in the late morning) and ending 3.5 h after the injection. The initial purpose of the 3.5 h collections was to measure urinary excretion rates of ^18^F-dopamine and related compounds; however, as the study progressed the specific activity of the administered tracer became too high to track these analytes reliably. Data about the endogenous catechols were obtained simultaneously and are the basis of the present report. There were insufficient data to assess potential differences from excretion rates on the basis of 24 h collections in the same subjects. The subjects’ diets were not controlled before the urine collections.

The urine was collected into pre-weighed plastic containers to which 30 mL of 6 N HCl had been added to ensure the urine was acidified. Urine volume was measured by weighing the container after the collection was complete and subtracting the weight of the pre-weighed container (the density of urine is essentially the same as that of water).

Patients were assigned to groups on the basis of previously published consensus statements [15, 18]. The group assignments were supplemented by results of olfactory testing and cardiac ^18^F-dopamine-derived radioactivity [35].

Urinary catechols were assayed in the laboratory of the Autonomic Medicine Section by batch alumina extraction followed by liquid chromatography with series electrochemical detection. Assay personnel were blinded as to the clinical and other laboratory data until the neurochemical results were tabulated for analysis.

When more than one urine collection was obtained in a given participant, the results were averaged across samples.

Data were excluded from the analysis in the event the plasma DOPA concentration was above the upper limit of normal in our laboratory, 2500 pg/mL (12.7 pmol/mL), which could indicate levodopa-carbidopa treatment. Data from patients on droxidopa at the time of study were also excluded from the analysis. Since droxidopa is a catechol, detection of high plasma or urine concentrations of droxidopa was straightforward by inspection of the chromatographs. Data from patients on medications known to inhibit neuronal uptake of catecholamines were also excluded.

Statistical analyses and graphics were done using GraphPad Prism for Mac OS 10.1.0 (GraphPad Software, LLC, Boston, MA). For comparisons of PD, PAF, and MSA groups versus the control group, one-way factorial analyses of variance were used with Dunnett’s post hoc test. Scatter plots were analyzed by linear regression, with calculation of Pearson correlation coefficients. The cutoff value for low ^18^F-dopamine-derived radioactivity was 6000 nCi-kg/cc-mCi [22]. A *p* value less than 0.05 defined statistical significance.

## Results

Data were reviewed for all the timed urine collections obtained in conjunction with ^18^F-dopamine PET at the NIH Clinical Center between 1994 and 2024.

After exclusion from analysis of all data from patients on levodopa-carbidopa, droxidopa, or drugs known to inhibit the cell membrane norepinephrine transporter, statistical analyses were conducted for 20 patients with PD, 19 patients with PAF, 16 patients with MSA, and 17 controls. Mean values and age ranges in the PD, PAF, MSA, and control groups were 63 (42–75), 63 (30–78), 59 (45–70), and 57 (22–75) years.

**Fig. 1:**
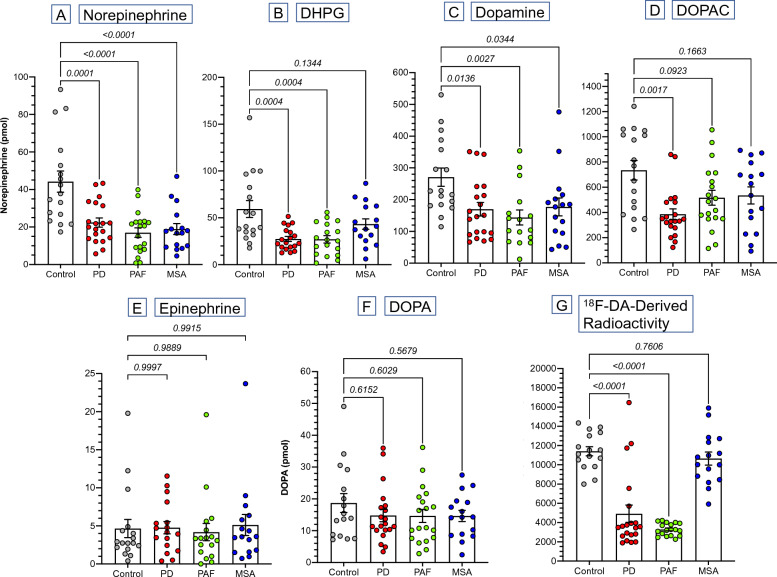
Individual values for urinary excretion rates of catechols (A-F) and for myocardial ^18^F-dopamine- (^18^F-DA)-derived radioactivity (**G**) in a control group (gray) and patient groups with Parkinson disease (PD, red), pure autonomic failure (PAF, green), and multiple system atrophy (MSA, blue). **A**–**G** show individual values and means ± standard error of the mean (SEM) for **A** norepinephrine, **B** 3,4-dihydroxyphenylglycol (DHPG), **C** dopamine, **D** 3,4-dihydroxyphenylacetic acid (DOPAC), **E** epinephrine (EPI), **F** 3,4-dihydroxyphenylalanine (DOPA), and **G** interventricular septal ^18^F-DA-derived radioactivity. Numbers in italics are *p* values for comparisons versus control on the basis of Dunnett’s post hoc test. PD, PAF, and MSA entailed decreased urinary norepinephrine and dopamine excretion with normal DOPA and EPI excretion. The PD and PAF groups also had decreased urinary DHPG excretion, whereas the MSA group did not, and the PD group had decreased urinary DOPAC excretion, whereas the MSA and PAF groups did not. ^18^F-DA-derived radioactivity highly efficiently separated the PD and PAF groups from the MSA and control groups

Urinary norepinephrine excretion was below control in PD (*p* = 0.0001), PAF (*p* < 0.0001), and MSA (*p* < 0.0001), while 3,4-dihydroxyphenylglycol (DHPG) was decreased in PD (*p* = 0.0004) and PAF (*p* = 0.0004) but not in MSA (*p* = 0.1344) (Fig. 1A and B).

The PD, PAF, and MSA groups also had decreased excretion rates of dopamine (*p* = 0.0136, 0.0027, 0.0344; Fig. 1C). DOPAC excretion was decreased in PD (*p* = 0.0017) but not in PAF or MSA (Fig. 1D). The groups did not differ in excretion rates of epinephrine (Fig. 1E) or DOPA (Fig. 1F).

The PD and PAF groups had low myocardial concentrations of ^18^F-dopamine-derived radioactivity compared with the control group (*p* < 0.0001 each), whereas the MSA group did not (Fig. 1F).

**Fig. 2:**
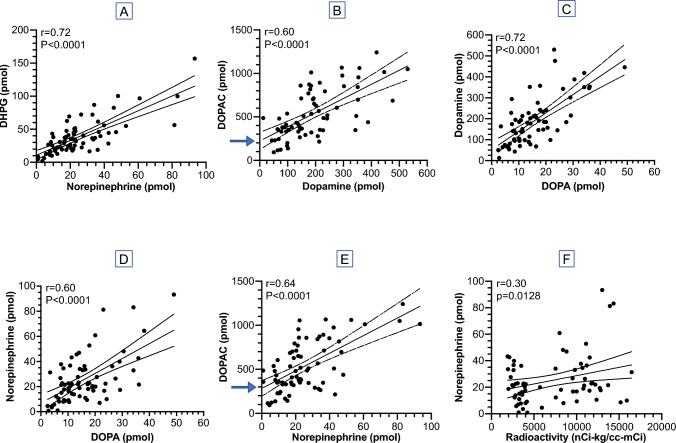
Correlations among urinary excretion rates of catechols across groups of patients with autonomic synucleinopathies and control subjects. Linear regression lines of best fit (solid lines) with 95% confidence intervals (dashed lines), correlation coefficients (*r*), and *p* values for **A** 3,4-dihydroxyphenylglycol (DHPG) versus norepinephrine, **B** 3,4-dihydroxyphenylacetic acid (DOPAC) versus dopamine, **C** dopamine versus DOPA, **D** DOPAC versus norepinephrine, and **E** norepinephrine versus cardiac ^18^F-dopamine-derived radioactivity. Urinary excretion rates of catechols were strongly positively correlated. Norepinephrine excretion was weakly positively correlated with ^18^F-dopamine-derived radioactivity

Across all participants, individual values for DHPG urinary excretion rates were strongly positively correlated with those for norepinephrine (Fig. 2A), DOPAC with dopamine (Fig. 2B), dopamine with DOPA (Fig. 2C), norepinephrine with DOPA (Fig. 2D), and DOPAC with norepinephrine (Fig. 2E). Similar positive correlations were obtained within each of the four subject groups (Supplementary Figs. 1–4). The regression lines of best fit generally passed close to the origin, except for those for DOPAC versus norepinephrine and for DOPAC versus dopamine, where the lines of best fit intersected the *y*-axis above the origin (arrows in Fig. 2B and E).

**Fig. 3:**
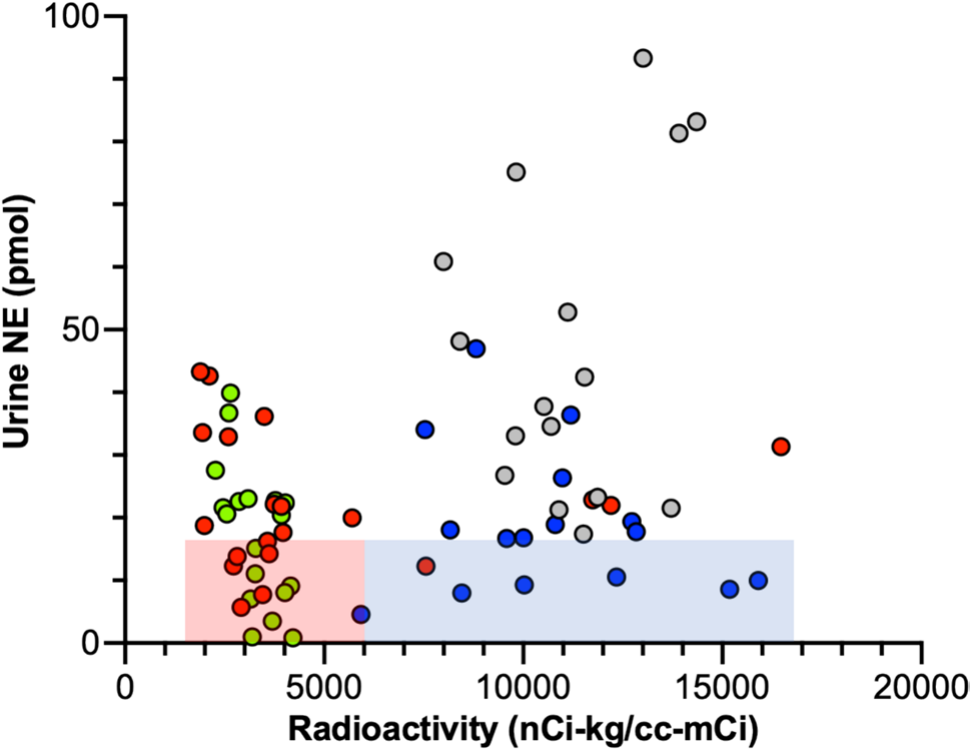
Individual values for urinary norepinephrine (NE) excretion and interventricular septal myocardial ^18^F-dopamine-derived radioactivity in patients with Parkinson’s disease (PD, red), pure autonomic failure (PAF, green), or multiple system atrophy (MSA, blue) and in control subjects (gray). Rectangles placed manually to highlight low urinary NE excretion with low myocardial ^18^F-dopamine-derived radioactivity in PD and PAF (pink) and low urinary NE excretion with normal myocardial radioactivity in MSA (blue).

Urinary norepinephrine excretion rates were weakly positively correlated with interventricular septal myocardial concentrations of ^18^F-dopamine-derived radioactivity (Fig. 2F). Among 15 synucleinopathy patients with norepinephrine excretion below the range of control and ^18^F-dopamine-derived radioactivity below the cutoff value of 6000 nCi-kg/cc-mCi (pink rectangle in Fig. 3), 14 had PD or PAF (pink rectangle in Fig. 3). Most patients with MSA had normal radioactivity. Within the MSA group there were five patients who had urinary norepinephrine excretion below the normal range and had normal cardiac ^18^F-dopamine-derived radioactivity.

## Discussion

In this observational, retrospective study we found that, compared with a control group, patient groups with PD, PAF, and MSA had decreased urinary excretion rates of norepinephrine and dopamine. These results add to evidence that in autonomic synucleinopathies catecholaminergic abnormalities are not confined to the brain. Most research attention has focused on splanchnic organs [9, 46] and the heart [48]; the present data extend to the kidneys the notion of peripheral catecholaminergic abnormalities in these diseases.

The finding that PD patients had decreased urinary excretion rates of dopamine and its deaminated metabolite DOPAC confirms a report from more than a half century ago [40]. We were unable to find previous literature on urinary excretion rates of these catechols in PAF or MSA.

Individual values for urinary excretion rates of norepinephrine, dopamine, DHPG, DOPAC, and DOPA were strongly positively correlated with each other. The linear regression lines of best fit passed close to the origin, suggesting a common source. Since DOPA is the precursor of the catecholamines, it is reasonable to infer that the shared source was DOPA.

**Fig. 4:**
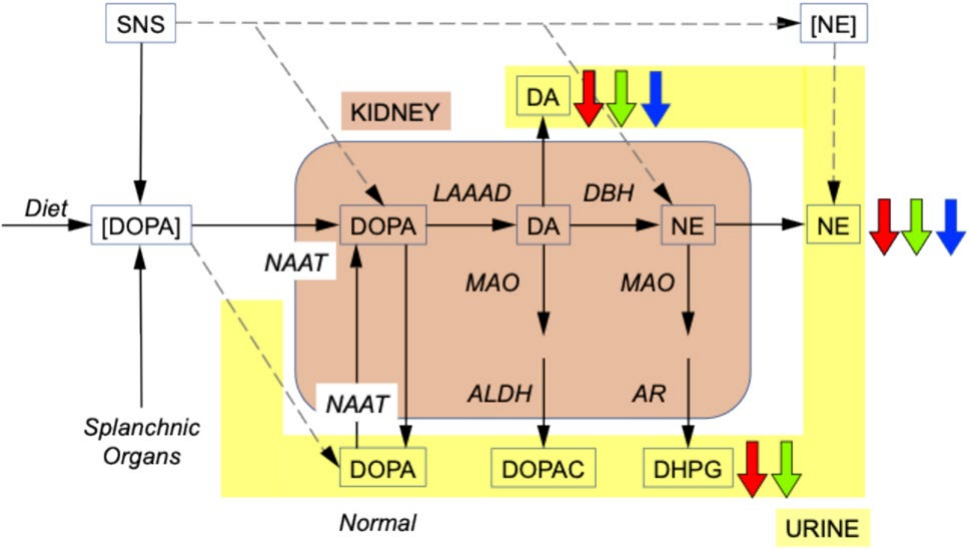
Concept diagram depicting sources of catechols in urine. Urinary norepinephrine (NE) excretion is derived from post-ganglionic nerves of the sympathetic noradrenergic system (SNS), glomerular filtration of circulating norepinephrine (NE), and dopamine (DA) via the enzyme dopamine-beta-hydroxylase (DBH). Urinary DA excretion is derived from proximal tubular cell uptake of circulating DOPA (DOPA) or from DOPA in the glomerular filtrate via neutral amino acid transporters (NAAT) followed by enzymatic decarboxylation via L-aromatic-amino-acid decarboxylase (LAAAD). DOPA is influenced by dietary factors, release from SNS nerves, and tyrosine hydroxylase expressed in splanchnic organs. Cytoplasmic DA is converted to the acid 3,4-dihydroxyphenylacetic acid (DOPAC) via monoamine oxidase (MAO) and aldehyde dehydrogenase (ALDH), and NE is converted to the glycol 3,4-dihydroxyphenylglycol (DHPG) via MAO and aldehyde/aldose reductase (AR). In general, urinary catechol excretion reflects a convergence of the SNS and the DOPA-DA autocrine-paracrine system. Not shown are urinary DOPAC and epinephrine derived from the circulation. The autonomic synucleinopathies Parkinson’s disease (PD, red arrows), pure autonomic failure (PAF, green arrows), and multiple system atrophy (MSA, blue arrows) were associated with decreased urinary excretion of NE and DA. PD and PAF were also associated with decreased urinary excretion of DHPG, but MSA was not

As indicated by the concept diagram in Fig. 4 renal DOPA has multiple potential determinants, including proximal tubular cell uptake of DOPA from the vasa rectae and glomerular filtrate via neutral amino acid transporters (NAATs) and DOPA derived from local sympathetic nerves. The present data cannot separate these mechanisms.

An exception to the lines of best fit passing through the origin seemed to obtain for urinary DOPAC excretion. From analysis of the scatterplots in Fig. 2B and E the regression lines of best fit for DOPAC versus dopamine and DOPAC versus norepinephrine were above the origin. There is a substantial amount of DOPAC in the circulation, and as an acid DOPAC is actively secreted from cells [41]. Theoretically, because of the relatively high concentration of DOPAC in the glomerular filtrate some DOPAC could be excreted even in the absence of local dopamine or norepinephrine production.

Urinary DHPG excretion was decreased in PD and PAF but not in MSA. Since LBDs involve in vivo and postmortem evidence for decreased renal norepinephrine content, whereas MSA does not [42, 55], low urinary norepinephrine excretion in MSA might reflect decreased exocytotic release owing to a pre-ganglionic sympathetic noradrenergic lesion rather than to a lesion of post-ganglionic nerves supplying the kidneys. The idea of a pre-ganglionic lesion in MSA fits with low cardiac ^18^F-dopamine-derived radioactivity in PD and PAF and normal radioactivity in most patients with MSA [26].

Several patients with MSA with normal cardiac radioactivity had urinary norepinephrine excretion rates below the range of controls (blue rectangle in Fig. 3). These results can be interpreted by decreased release of norepinephrine from intact renal sympathetic nerves. Since patients with MSA have normal spillover of norepinephrine into the cardiac venous drainage [20], there might be differences in renal compared to cardiac sympathetic noradrenergic function in MSA.

Considering that virtually all of urinary dopamine in humans is derived from circulating DOPA [59], the results lead us to propose that autonomic synucleinopathies entail abnormal functioning of the renal DOPA-dopamine autocrine-paracrine system [24]. We speculate that low urinary excretion rates of dopamine and DOPAC in PD reflect decreased local activity of L-aromatic-amino-acid decarboxylase (LAAAD), the enzyme that catalyzes the conversion of DOPA to dopamine, or decreased renal uptake of circulating DOPA via NAATs. LAAAD is well known to be very abundant in the kidneys, and pharmacological inhibition of the enzyme markedly decreases the concentration ratio of dopamine/DOPA in urine [17]. Moreover, α-syn inhibits LAAAD [54], a finding that could link renal catecholaminergic abnormalities with synucleinopathy. Finally, a recent proteomic study of biofluids including urine has identified LAAAD as a biomarker of early PD [51].

### Limitations

A major limitation in interpreting results of this study is the gap in knowledge about the relative contributions of renal sympathetic innervation and the DOPA-dopamine autocrine-paracrine system to urinary catecholamine excretion in humans (see the concept diagram in Fig. 4). Technologies exist that could fill these gaps. For instance, sympathetic noradrenergic innervation of the kidneys can be visualized and quantified by PET using the imaging agent ^11^C-methylreboxetine, which is a ligand for the cell membrane norepinephrine transporter [31]. The sequence of renal uptake of circulating DOPA and intracellular LAAAD activity could be assessed by measuring plasma levels and urinary excretion rates of norepinephrine after dosing with the norepinephrine pro-drug droxidopa (L-threo-3,4-dihydroxyphenylserine, L-DOPS, Northera®). If there were attenuated renal uptake or enzymatic decarboxylation of neutral amino acids in autonomic synucleinopathies, then the urinary concentration ratio of norepinephrine/droxidopa would be decreased.

In conclusion, the present results indicate low urinary excretion rates of catecholamines in autonomic synucleinopathies of systemic and local abnormalities. The pathophysiologic bases for decreased urinary excretion of dopamine and norepinephrine in PD, PAF, and MSA are unknown but may include abnormalities of the renal sympathetic noradrenergic system, the renal DOPA–dopamine autocrine-paracrine system, or both systems. Future studies could pinpoint the underlying mechanisms.

## Supplementary Information

Below is the link to the electronic supplementary material.

Supplementary file1 (DOCX 504 KB)

Supplementary file2 (XLSX 152 KB)

## Data Availability

The data for this report are in the Supplementary Data Spreadsheet.
