# Supplementary material for: Decreased urinary excretion of norepinephrine and dopamine in autonomic synucleinopathies
Source: Clin Auton Res. 2024 Dec 10;35(2):215–22. doi: 10.1007/s10286-024-01093-6 (PMC12000174; doi:10.1007/s10286-024-01093-6)
Supplement: Supplementary file 1 — Supplementary file1 (DOCX 504 KB) [file 10286_2024_1093_MOESM1_ESM.docx]

**Supplementary Fig. 1: Individual values for urinary 3,4-dihydroxyphenylglycol (DHPG) and norepinephrine excretion in patients with Parkinson’s disease PD, red), pure autonomic failure (PAF, green), or multiple system atrophy (MSA, blue) and in control subjects (gray).**

Linear regression lines of best fit (solid lines) with 95% confidence intervals (dashed lines) are also shown. DHPG excretion was positively correlated with norepinephrine excretion.

**Supplementary Fig. 2: Individual values for urinary 3,4-dihydroxyphenylacetic acid (DOPAC) and dopamine excretion in patients with Parkinson’s disease PD, red), pure autonomic failure (PAF, green), or multiple system atrophy (MSA, blue) and in control subjects (gray).**

Linear regression lines of best fit (solid lines) with 95% confidence intervals (dashed lines) are also shown. DOPAC excretion was positively correlated with dopamine excretion.

**Supplementary Fig. 3: Individual values for urinary norepinephrine and dopamine excretion in patients with Parkinson’s disease PD, red), pure autonomic failure (PAF, green), or multiple system atrophy (MSA, blue) and in control subjects (gray).**

Linear regression lines of best fit (solid lines) with 95% confidence intervals (dashed lines) are also shown. Norepinephrine excretion was positively correlated with dopamine excretion.

**Supplementary Fig. 4: Individual values for urinary norepinephrine and DOPA excretion in patients with Parkinson’s disease PD, red), pure autonomic failure (PAF, green), or multiple system atrophy (MSA, blue) and in control subjects (gray).**

Linear regression lines of best fit (solid lines) with 95% confidence intervals (dashed lines) are also shown. Norepinephrine excretion was positively correlated with DOPA excretion.
